# Pursuing novel molecular mechanisms and treatment strategies to enhance lung growth in neonatal chronic lung disease (CLD)

**DOI:** 10.1186/2194-7791-2-S1-A30

**Published:** 2015-07-01

**Authors:** Miguel A Alejandre Alcazar, Mark Kaschwich, Stefanie Preuss, Robert Ertsey, Sana Mujahid, Eva Rother, Katharina Dinger, Jörg Dötsch, Marlene Rabinovitch, Richard Bland

**Affiliations:** Department of Pediatrics, Cardiovascular Institute, Stanford University, Stanford, CA USA; Department of Pediatrics and Adolescent Medicine, University of Cologne, Germany

## Rationale

Infants born prematurely or with intrauterine growth restriction (IUGR) are often afflicted with CLD, a debilitating disorder marked by diminished alveoli and lung micro-vessels. Despite advances in neonatology, CLD's incidence remains high and its prevention elusive. Mechanical ventilation (MV), high inspired O_2_, and IUGR can disrupt key signaling pathways, reduce cell survival and disrupt matrix elements, thus inhibiting lung growth [1–6].

## Aims

**(1)** To define the impact of MV, O_2_ and IUGR on lung structure and function in newborn rodents.

**(2)** identify altered signaling pathways of neonatal lung injury, and test novel treatment strategies.

**(3)** mechanistic studies to define how such aberrant signaling may impact cell survival in culture.

## Methods

**(1)** Exposure of newborn mice to prolonged MV or hyperoxia; IUGR in rats induced by maternal low protein diet.

**(2)** Prolonged MV-O_2_ in pups treated with a serine elastase inhibitor Elafin.

**(3)** Studies with mouse alveolar epithelial (AEC) and lung micro-vascular endothelial (LMVEC) cells.

## Results

MV, O_2_, and IUGR inhibit alveolar and micro-vessel formation and disrupt matrix components in lung, resulting in reduced respiratory system compliance and increased airway resistance. TGFβ signaling is disrupted after MV, O_2_, and IUGR which adversely affects homeostasis of AEC and LMVEC, contributing to impaired lung growth with disordered lung matrix. Moreover, MV-O_2_ inhibited EGF-receptor activation and reduced Krüppel-like factor 4 (Klf4), a regulator of cell differentiation and survival. We identified this novel pEGFR-Klf4 axis as a key regulator of AEC survival, and discovered that pEGFR-Klf4 signaling is protected by treatment with Elafin during MV-O_2_.

## Conclusion

These results identify critical pathways that can impact lung growth arrest, as seen in CLD, and uncover novel mechanisms that could promote alveolarization, thus pointing the way toward new treatments to improve the outcomes of infants with CLD.
